# How active and passive social media use affects impulse buying in Chinese college students? The roles of emotional responses, gender, materialism and self-control

**DOI:** 10.3389/fpsyg.2022.1011337

**Published:** 2022-09-30

**Authors:** Si Chen, Kuiyun Zhi, Yongjin Chen

**Affiliations:** ^1^School of Business and Administration, Chongqing Technology and Business University, Chongqing, China; ^2^School of Public Policy and Administration, Chongqing University, Chongqing, China

**Keywords:** social media use, impulse buying, emotional responses, college students, China

## Abstract

Social media plays a vital role in consumers’ purchasing decision making. There are still gaps in existing research on the relationship between divided dimensions of social media use and impulse buying, as well as the mediating and moderating effects therein. This study explored the mediation and moderation effects in the relationship between different social media usage patterns, emotional responses, and consumer impulse buying. Data from 479 college students who were social media users in China were analyzed using structural equation modeling. The results showed that active and passive social media use were significantly and positively associated with users’ enjoyment, whereas passive social media use significantly increased depression. Both enjoyment and depression were significantly and positively associated with users’ impulse buying. Materialism positively moderated the relationship between enjoyment and impulsive consumption, while self-control significantly reduced the effect of depression on impulse buying. These findings that emotion mediated and personality traits moderated relationships between social media use and impulse buying expand impulsive purchase literature and provide insights for guiding college students’ healthy use of social media and rational consumption.

## Introduction

With the continuous development of mobile internet, especially during the COVID-19 pandemic, social media (e.g., Facebook and YouTube) encompasses a broad range of online venues that facilitate communication, interaction, and the exchange of content among users (Kim and Johnson, 2016; Wang P. et al., 2020). In addition to its social feature, social media also offers commercial features, which have created a new e-commerce model known as social commerce (Van Tran et al., 2022). For example, social media provides a platform for businesses to attract consumers and promote products, and for consumers to obtain suggestions and share experiences (Leong et al., 2018). Through new ways of marketing (e.g., word-of-mouth marketing, brand marketing, and influencer marketing), social media has greatly changed business marketing strategies and consumer purchase decision-making processes (Kim and Johnson, 2016; Guo and Li, 2022). Several studies have demonstrated that social media significantly influences consumer behavior (Kim and Johnson, 2016; Zafar et al., 2020, 2021), but the purchase decision-making process of social media users with different usage patterns has been relatively ignored. It has been claimed that 30 to 50% of purchase decisions are impulsive purchases, and that 84% of all shoppers have bought items impulsively (Husnain et al., 2019; Zafar et al., 2021). Therefore, the main concern of this study is to explore how social media usage patterns affect users’ impulse buying behavior.

A few scholars have mainly focused on the direct relationship between social media use and impulse buying (Leong et al., 2018; Lahath et al., 2021; Pellegrino et al., 2022). Pellegrino et al. (2022) found a direct relationship between social media intensity and impulsive consumption behavior. Similarly, Lahath et al. (2021) study also confirmed the positive direct impact of social media use on impulse buying during the COVID-19 pandemic. Leong et al. (2018) investigated the influence of Facebook usage intensity on F-commerce impulse buying and the mediating role of urge to purchase. However, the existing literature lacks specific studies on the impact of different social media usage patterns on impulse buying and other mediating variables, such as emotion.

Research shows that the two most common patterns of social media use refer to active and passive use (Burke et al., 2010; Chen, 2021). Active use includes posting, liking, commenting on content, and interacting with others on social media, whereas passive use refers to browsing others’ posts or content shared by friends without any liking, commenting, or interacting (Verduyn et al., 2017). Previous empirical studies have reported that active social media use increases users’ social connection and social support, and enhances their positive emotion and well-being, whereas passive social media use increases users’ negative emotions, such as upward social comparison, envy, depression, and anxiety (Chen S. et al., 2019; Valkenburg et al., 2021, 2022). Accordingly, active and passive social media use induce different emotional responses. It has been shown that emotions play a vital role in shaping impulse purchases, whether positively or negatively (Verplanken et al., 2005; Liu et al., 2019; Zhao et al., 2021). Therefore, active and passive social media use may impact users’ impulse purchases through different mediating effects of positive or negative emotions. The outbreak of COVID-19 has led to a rapid growth of individuals’ social media use, greater emotional ups and downs, and exacerbated depression and anxiety (Wang C. et al., 2020; Luo et al., 2021b), which cause increased impulsive consumption (Lahath et al., 2021). However, to the authors’ best knowledge, no study has examined how active and passive social media use influence users’ impulse buying behavior, and the emotional mediation therein. Past research indicates that gender, materialism, and self-control play key roles in impulsive consumption decision-making (Isabelle, 2016; Li et al., 2020), but how these factors moderate the relationship between social media use and impulsive consumption is still unknown.

The above evidence suggests that the current understanding of the relationship between social media usage patterns and impulse buying, as well as the moderating and emotional mediating effects therein, is limited. To fill this gap, we developed a model drawing on social cognitive theory (SCT) to explore the relationship between active and passive social media use, different emotions (e.g., enjoyment and depression), and impulse buying behavior. The moderating effects of gender, materialism, and self-control were also examined. The next section reviews the related literature and develops hypotheses. Section 3 provides the research method, and section 4 presents the results of the data analysis. The conclusion and discussion are elaborated in section 5. In the last section, implications and limitations are illustrated.

## Theoretical background and hypotheses

### Social cognitive theory

**Figure 1:**
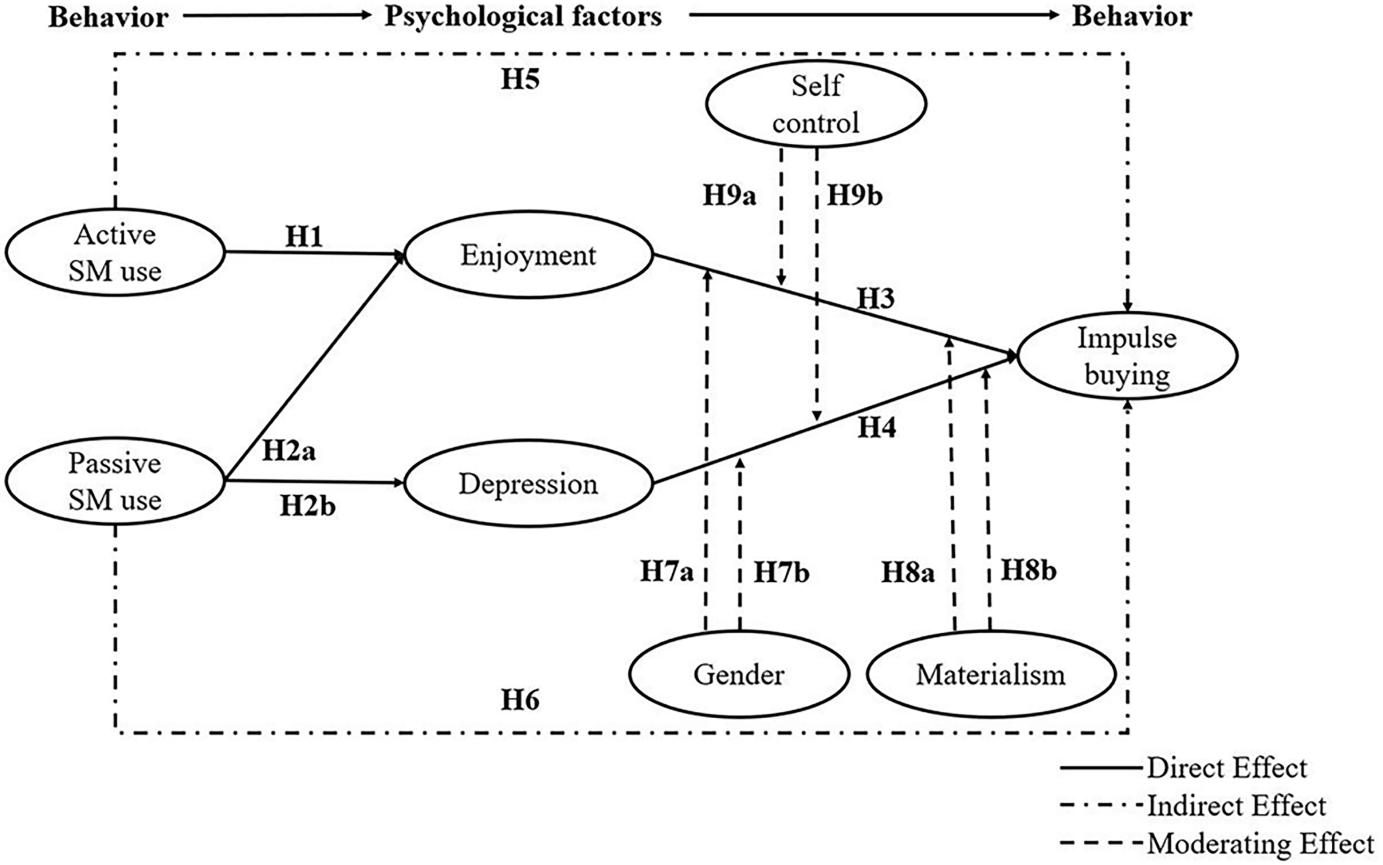
Theoretical model.

Social cognitive theory was first proposed by Bandura (1986), and proposes that there is a triadic reciprocity relationship between an individual, the environment, and behavior. Specifically, this triadic reciprocity relationship refers to the interaction between an individual’s behavior, psychology, or cognition and their environment (Mohamed and Ahmad, 2012). This theory provides a useful framework for explaining the complex relationships between users’ psychological and environmental factors and behavior in social media. For example, Yoo and Jeong (2017) found a reciprocal interaction between social media use and depression. Moreover, Chen S. et al. (2019) study showed that social network use affects users’ mood, well-being, and social capital, which in turn predict their continued use intention. According to social support theory, active social media use could boost social support and positive emotions (Frison and Eggermont, 2016). Based on social comparison theory, passive browsing of social media content could exacerbate negative emotions (Tandoc et al., 2015). The Stimuli-Organism-Response model indicates that emotional stimuli are the key factors predicting consumer behavior (Zhao et al., 2021). Given that SCT highlights the reciprocal effects between behavioral and psychological factors, it could serve as the theoretical framework for this study to investigate the relationships between social media use, emotion, and impulse buying (see Figure 1).

### Active and passive social media Use

Active social media use refers to the behavioral pattern of actively creating content or interacting with others; specific behaviors include posting updates and photos, sharing information, meeting new people online and chatting with them, and participating in groups (Shaw et al., 2015). Passive social media use refers to the behavioral pattern of only browsing and consuming information, such as browsing friends’ statuses or personal homepages (Ding et al., 2017). Active use contributes content and relational resources to the social media platform, while passive use does not (Chen et al., 2014).

The contradictory findings on the impact of general social media use on psychological outcomes may be because scholars have failed to consider the different ways in which users use social media, namely, active and passive use (Verduyn et al., 2017). Several studies that have taken this difference into account have found that individuals’ active social media use usually has a positive impact on their psychological factors, and, conversely, that passive social media use usually has a negative impact on psychological factors (Matook et al., 2015; Frison and Eggermont, 2016, 2020). Thus, active and passive social media use could lead to different emotions and behaviors.

### Impulse buying

Although previous studies have adopted different definitions of impulse buying, they agree that impulse buying has three characteristics. First, it is an unplanned behavior (Stern, 1962; Li et al., 2020; Lina et al., 2022). Second, it is an impulsive reaction. Consumers will react impulsively when they are affected by external stimuli, and this impulsive response can generate new purchase needs or stimulate potential purchase needs that manifest as strong, sudden, or accidental impulses (Rook, 1987; Parboteeah et al., 2009; Xiang et al., 2016). Third is emotional factors. Impulse buying is often caused by strong emotional factors that can make consumers temporarily irrational, which results in impulse buying behavior (Liu et al., 2019).

Previous work has concentrated on the determinants of impulsive consumption in social commerce according to environmental stimuli, such as platform-related factors, social-related factors, and marketing-related factors (Chen Y. et al., 2019; Liu et al., 2019; Abdelsalam et al., 2020; Zafar et al., 2021; Yu, 2022). Consumer characteristics such as arousal, pleasure, and urgency have often been regarded as mediating variables in the relationship between environmental stimuli and impulse buying (Abdelsalam et al., 2020). The few studies on social media use and impulse buying have largely confirmed the presence of a direct effect of social media use intensity on consumers’ impulse buying (Leong et al., 2018; Lahath et al., 2021; Pellegrino et al., 2022). Moreover, one study found no direct correlation between passive browsing and impulse buying, but that the urge to buy fully mediated passive browsing and impulse consumption (Leong et al., 2018). However, the relationship between active and passive social media use, emotion, and impulse buying remains unclear.

### Active and passive social media use and emotion

Active social media use, such as interacting with others, can help increase users’ immediate positive emotions, thereby increasing their perceived social support and improving their life satisfaction in the long-term (Kross et al., 2013; Oh et al., 2014). Through interaction and communication on social media, people can also experience more happiness and excitement (Zhang and Leung, 2015). According to Ekman (1992), enjoyment is one of the seven basic emotions. Enjoyment has been defined as a pleasurable emotional response to an enjoyment-inducing environment (Valkenburg et al., 2021). Active social media use also has a significant positive effect on users’ positive emotion and sense of social connection (Verduyn et al., 2017), which can be enjoyable for them. It is worth mentioning that numerous (36) cross-sectional and longitudinal studies found no significant association between active social use and depression (Valkenburg et al., 2022). Considering this existing empirical evidence, we expected to see no significant correlation between active social media use and depression. Based on the above findings, we made the following hypothesis:

*H1*: Active social media use is positively associated with users’ enjoyment.

That said, there is strong evidence that passive social media use can often impair users’ mental health, such as increased depression, anxiety, and loneliness, and decreased subjective well-being (Krasnova et al., 2015; Shaw et al., 2015). However, this does not necessarily mean that browsing social media has negative psychological outcomes. For example, Lin and Utz (2015) demonstrated that users may experience more positive emotions when browsing Facebook. The authors also observed the phenomenon of “emotional contagion” in social media, whereby individuals tend to experience positive emotions when browsing posts containing positive emotions. Valkenburg et al. (2021) also confirmed this finding in adolescents, whereby browsing-induced enjoyment had positive effects. Accordingly, we made the following hypothesis:

*H2a*: Passive social media use is positively associated with users’ enjoyment.

Depression can be defined as an emotional state in which an individual feels sad and gradually loses interest in engaging in activities, and this is usually accompanied by many negative emotions, such as disgust, frustrated, and annoyance (Yoo and Jeong, 2017). According to social comparison theory, individuals usually compare themselves with similar others when they evaluate themselves (Festinger, 1954). Social media has become an important channel for individuals to present themselves positively (Tandoc et al., 2015). When browsing others’ generated content on social media, consumers are likely to encounter positive self-presentations of other people, which may lead to lurkers’ social comparison and envy (Frison and Eggermont, 2016; Valkenburg et al., 2021). Users may believe that other people live happier lives than themselves, which could cause an exacerbation in depression (Chen S. et al., 2019; Valkenburg et al., 2022). Based on these previous findings, we made the following hypothesis:

*H2b*: Passive social media use is positively associated with users’ depression.

### Emotion and impulse buying

According to Rook (1987), impulse buying could be considered as an irrational consumption behavior characterized by low cognitive assessment and high emotional arousal. Previous research has confirmed that pleasure is a key emotional factor driving impulse buying (Zhao et al., 2021). Shen and Khalifa (2012) found that a delighted emotional experience has a positive effect on subsequent buying behavior. Moreover, Xiang et al. (2016) demonstrated that users who feel that social commerce usage is pleasing and enjoyable are more likely to buy items on impulse. Enjoyment can be defined as a pleasurable emotional response (Valkenburg et al., 2021). When users feel pleasant while on a social media platform, they are likely to have the urge to engage in impulse buying. Therefore, we made the following hypothesis:

*H3*: Enjoyment is positively associated with social media users’ impulse buying.

Several studies have reported that impulsive consumption can be fueled by negative affect (Verplanken et al., 2005; Liu et al., 2019). Indeed, consumers with more negative emotions during the COVID-19 pandemic reported more frequent daily impulse purchases (Xiao et al., 2020). This can be explained by the “mood congruency effect” (Cunningham, 1988). According to this effect, when experiencing negative emotions, an individual’s thoughts and judgments are often distorted towards being more negative. Furthermore, people with negative emotions process information less systematically when making judgments and decisions (Raghunathan and Pham, 1999). Previous work has shown that consumers may consider impulsive buying as a way to escape negative emotions (Silvera et al., 2008). Considering that depression is often accompanied by negative emotions, such as sadness, frustration, annoyance, consumers who feel depressed while using social media may view impulse buying behavior as a form of self-soothing that alleviates negative emotion and makes them feel better. Thus, we hypothesized the following:

*H4*: Depression is positively associated with social media users’ impulse buying.

### The mediation effect of enjoyment and depression

Several studies have reported the mediating role of enjoyment in the relationship between external stimuli and behavioral intention, such as purchase or electronic-word-of-mouth intention (Ukpabi and Karjaluoto, 2018). In addition, depression has been reported to be a mediator in the relationship between users’ different social media use and their subsequent behaviors (Chen S. et al., 2019). Following SCT, there is a mutual interaction between an individual’s emotion or cognition and his/her behavior (Mohamed and Ahmad, 2012). Considering the phenomenon of emotional contagion (Lin and Utz, 2015) and social comparison theory (Frison and Eggermont, 2016), active and passive social media use is positively associated with enjoyment, whereas passive social media use positively predicts depression. According to the emotional stimulation effect of the stimulus-organism-response model (Zhao et al., 2021) and the mood congruency effect (Cunningham, 1988), enjoyment and depression positively predict consumers’ impulse buying behavior. Thus, based on the above inferences (H1-H5) and previous research findings, it is possible that enjoyment and depression mediate the relationship between consumers’ social media use and their impulse purchasing behavior. We therefore proposed the following hypotheses:

*H5*: Enjoyment mediates the association between active social media use and impulse buying.

*H6a*: Enjoyment mediates the association between passive social media use and impulse buying.

*H6b*: Depression mediates the association between passive social media use and impulse buying.

### The moderation of gender, materialism, and self-control

With regard to gender, recent studies have shown that women have stronger impulse buying tendencies and are more prone to making impulse purchases than men (Segal and Podoshen, 2013; Styvén et al., 2017). This is not only the case in adults; female adolescents are also more inclined to be impulsive buyers than male adolescents (Isabelle, 2016). One reason for this is that girls are higher scored on all measures of emotional states than boys, underlying a more emotional background for girls than for boys (Moksnes et al., 2010; Isabelle, 2016). That is, female users are more likely to have higher positive or negative emotional states and thus are more likely to have impulse buying tendencies than male users. This is consistent with previous research showing that female consumers may exhibit stronger impulse buying tendency than male consumers, when influenced by emotions (Coley and Burgess, 2003). In addition, women are more likely to buy to manage their emotions or compensate for their bad emotions than men (Coley and Burgess, 2003; Ching et al., 2016). Based on the above, we made the following hypothesis:

*H7a*: Gender moderates the relationship between enjoyment and social media users’ impulse buying. Specifically, for female users, enjoyment has a greater impact on their impulse buying than it does for male users.

*H7b*: Gender moderates the relationship between depression and social media users’ impulse buying. Specifically, for female users, depression has a greater impact on their impulse buying than it does for male users.

The effect of the personal trait of materialism on impulse buying behavior has been increasingly emphasized by scholars (Pellegrino et al., 2022). Materialism refers to a value that emphasizes the importance of having material wealth in one’s life (Richins and Dawson, 1992). This value highlights personal value and meaning in life through the possession of material wealth, thereby enhancing well-being (Kilbourne and Pickett, 2008). Prior research has suggested that individuals with high materialism tend to evaluate their success by the quantity and quality of purchased goods or services, which leads to more impulsive buying behaviors (Podoshen and Andrzejewski, 2012; Pellegrino et al., 2022). Materialistic people buy goods impulsively because of the positive emotion this provides. For individuals with high materialism, the influence of emotions on impulse buying will be stronger. Based on the above evidence, we made the following hypotheses:

*H8a*: Materialism positively moderates the relationship between enjoyment and social media users’ impulse buying.

*H8b*: Materialism positively moderates the relationship between depression and social media users’ impulse buying.

Self-control refers to an individual’s ability to transcend or alter their internal responses, as well as interrupt undesired behavioral tendencies (such as impulsiveness) and restrain their behavior (Tangney et al., 2004). Self-control is one of the vital influencing factors of impulse buying. Rational buying behavior requires a struggle between self-control and the desire to buy impulsively (Nagar, 2016). If control is overwhelmed by desire, the consumer succumbs to the emotional push and product appeal and makes a purchase. When feeling out of control, an individual’s emotions accelerate to produce irrational and impulsive behavior, such as impulsive consumption (Loewenstein, 1996; Li et al., 2020). Therefore, social media users with low self-control are more likely to make impulse purchases due to emotional stimuli. Thus, the two following hypotheses were made:

*H9a*: Self-control negatively moderates the relationship between enjoyment and social media users’ impulse buying.

*H9b*: Self-control negatively moderates the relationship between depression and social media users’ impulse buying.

## Materials and methods

### Sample and data collection

The research model was tested using data collected from college students by employing a professional questionnaire platform through random sampling method. College students were chosen as the sample for three main reasons. First, compared to other age groups, young people are more impulsive (Pechmann et al., 2005). Second, young people are the main users of social media (Van Tran et al., 2022). College students who grew up in the Internet age are more inclined to use social media to communicate than to engage in face-to-face communication (Punyanunt-Carter et al., 2018). Third, young people are the main force of online shopping (Van Tran et al., 2022).

Given the prevention and control policies in place during the COVID-19 pandemic in China, we conducted an online survey through the Credamo platform. Credamo has a sample pool of more than 2.8 million members from diverse backgrounds. The questionnaire was randomly sent to college students on Credamo from June to July, 2022. All respondents were informed of the objectives of the study, the anonymity of their answers, were told that participation was completely voluntary, and confirmed that their survey data could be transmitted to researchers. They continued to complete the questionnaire after giving their informed consent.

To ensure the quality of answers, we set up two attention screening questions and limited the IP and the number of answers (namely, participants with the same IP address could only complete the questionnaire once). Structural equation modeling was used to test our research model. A well-known “rule of 10,” as reported in previous studies, states that structural equation modeling estimation requires at least 10 subjects for each indicator (Nunnally, 1967; Westland, 2010). A total of 523 respondents completed the survey questionnaire, and 44 invalid samples were excluded. Data from a final total of 479 undergraduate and graduate students were included, covering 110 prefecture-level cities in 29 provincial administrative regions in China. Considering the division groups of Chinese young people (CBNData, 2019), we divided the age of the subjects into three levels, the criteria of which were adult (above 18 years), post-00s (22 years and younger), and post-95 s (27 years and younger). Post-95 s are also known as Generation Z (Djafarova and Bowes, 2021). Of the 479 respondents, 50.7% were male, 49.3% were female, and 99.4% of them were Generation Z (27 years and younger). Most participants had a monthly expense of 1,000–2000 RMB (66.4%) and used social media for more than 2 h per day (69.2%) (see Table 1).

**Table 1:** Profile of the respondents.

Characteristics	Levels	Frequency	Percentage (%)
Gender	Male	243	50.7
	Female	236	49.3
Age	18–22	400	83.5
	23–27	76	15.9
	28–40	3	0.6
Education	Junior college	42	8.8
	Undergraduate	412	86
	Postgraduate	25	5.2
Expense per month	0 ~ 1,000 RMB	43	9.0
	1,000–2,000 RMB	318	66.4
	2000–3,000 RMB	89	18.6
	3,000–4,000 RMB	12	2.5
	4,000–5,000 RMB	6	1.3
	5,000–8,000 RMB	8	1.7
	8,000–10,000 RMB	2	0.4
	10, 000–20,000 RMB	1	0.2
Time spent on social media per day	<30 min	4	0.8
	30 min ~ 1 h	26	5.4
	1–2 h	118	24.6
	2–4 h	177	37.0
	>4 h	154	32.2

### Measures

The questionnaire contained two sections. The first section collected data on the demographics of participants (e.g., gender, age, and education), expenses per month, and the time spent on social media per day. The second section consisted of seven constructs that assessed active and passive social media use, depression, enjoyment, materialism, self-control, and impulse buying.

#### Independent variables

Active and passive social media use was assessed using the measurement scales developed by Gerson et al. (2017) and Chen S. et al. (2019). Active social media use was measured using 5 items (e.g., “Posting status updates”; see Appendix A), and passive social media use was measured using 2 items (e.g., “Browsing the postings on social media passively without liking or commenting on anything”). Three items (ASMU3, PSMU3, and PSMU4) were excluded to ensure reliability, because these item loadings were below 0.5 in the factor analysis. Participants responded to items on a 5-point scale ranging from 1 (never (0% of the time)) to 5 (very frequently (100% of the time)). The Cronbach’s α values of the active and passive social media use measures in this study were 0.784 and 0.779, respectively.

#### Dependent variable

The 3-item Impulsive Consumption Scale (Li et al., 2020) was used to assess impulsive buying behavior (e.g., “Recently, I often have the impulse to buy products that I did not intend to buy”; see Appendix A). Items were scored on a 5-point Likert ranging from 1 (strongly disagree) to 5 (strongly agree), for which the Cronbach’s α coefficient was 0.877.

#### Mediation variables

The measurement items of enjoyment were adapted from Heijden (2003) and Gan and Li (2018), and included 4 items (e.g., “Using social media is an agreeable way of passing time”; see Appendix A). Participants responded to items on a 5-point Likert scale ranging from 1 (strongly disagree) to 5 (strongly agree). The Cronbach’s α coefficient in this study was 0.82.

The measurement of depression was adapted from Tandoc et al. (2015) and Chen S. et al. (2019). The scale included 5 items (e.g., “I felt sad”; see Appendix A). Respondents were asked to report the frequency of depression symptoms in the last week. Items were scored on a 5-point scale ranging from 1 (not at all) to 4 (a lot). The Cronbach’s α coefficient of the depression scale in this study was 0.852.

#### Moderation variables

The measures of materialism were adapted from Richins and Dawson (1992) and Li et al. (2020). The scale included 5 items (e.g., “I’d be happier if I could afford to buy more things”; see Appendix A). The items were scored on a 5-point Likert scale ranging from 1 (strongly disagree) to 5 (strongly agree). The Cronbach’s α coefficient in this study was 0.823.

Four measurement items from the “impulse control” dimension of the Chinese version of the Brief Self-Control Scale were used to measure self-control (Luo et al., 2021a). All of these measures were reverse-scored (e.g., “Sometimes I cannot help but do things that I know are wrong”). Participants responded to items on a 5-point Likert scale ranging from 1 (strongly disagree) to 5 (strongly agree). The Cronbach’s α of the self-control measures in this study was 0.83.

#### Control variables

Two control variables that measured users’ characteristics were also included in this study: monthly expenditure and time spent on social media per day. The existing literature has demonstrated that monthly spending level is closely associated with consumer’s impulse buying behavior (Vohs and Faber, 2007). Moreover, some scholars have reported that the time spent on social media is correlated with users’ psychological factors, such as well-being, social comparison, and envy (Arampatzi et al., 2018; Van Tran et al., 2022).

## Results

This study used structural equation modeling algorithms to analyze the data and test the hypotheses. Following a two-step approach, we examined the measurement model and structural model sequentially. During this process, estimations performed using AMOS 24 and SPSS 25 software (IBM, United States).

### Measurement model

Both reliability and validity were fulfilled in estimating the measurement model. Reliability can be established using Cronbach’s alpha values and composite reliabilities. As shown above and in Table 2, the Cronbach’s α and composite reliability values of all constructs were greater than 0.7, which implies that the results obtained from the scale’s seven variables are reliable (Fornell and Larcker, 1981; Hair et al., 2009).

**Table 2:** Construct reliability and validity.

Constructs	AVE	CR	ASMU	PSMU	EN	DE	IB	MA	SC
ASMU	0.519	0.843	**0.720**						
PSMU	0.758	0.862	0.233	**0.871**					
EN	0.605	0.859	0.357	0.315	**0.778**				
DE	0.615	0.888	−0.061	0.136	−0.222	**0.784**			
IB	0.707	0.879	0.265	0.058	0.126	0.173	**0.841**		
MA	0.544	0.856	0.122	0.221	0.287	0.154	0.462	**0.738**	
SC	0.619	0.867	0.07	−0.131	0.055	−0.299	−0.371	−0.368	**0.787**

ASMU, active social media use, PSMU, passive social media use, EN, enjoyment, DE, depression, IB, impulse buying, MA, materialism, SC, self-control.

Validity can be established by convergent validity and discriminant validity. Average variance extracted values exceeded the suggested threshold value of 0.5 (see Table 2), and showed an adequate convergent validity (Fornell and Larcker, 1981; Hair et al., 2009). A good discriminant validity is verified in two ways. As shown in Table 2, the square roots of the average variance extracted values (diagonal elements in bold) are greater than the correlation coefficients between any two variables (non-diagonal elements) (Fornell and Larcker, 1981). Moreover, every within-construct item loads on the measured construct (bold values) higher than on the other constructs (Chin et al., 2003) (see Table 3).

**Table 3:** Factor loadings.

	ASMU	PSMU	EN	DE	IB	MA	SC
ASMU1	**0.730**	−0.066	0.125	0.007	0.080	−0.029	−0.060
ASMU2	**0.746**	−0.023	0.068	0.063	0.108	−0.009	0.063
ASMU4	**0.650**	0.078	0.081	−0.111	0.062	0.117	0.055
ASMU5	**0.708**	0.154	0.050	0.019	0.014	0.125	0.083
ASMU6	**0.762**	0.082	0.145	−0.063	0.065	−0.029	−0.015
PSMU1	0.144	**0.878**	0.097	0.085	0.016	0.085	−0.020
PSMU2	0.054	**0.863**	0.179	0.054	−0.022	0.073	−0.077
EN1	0.150	0.039	**0.781**	−0.116	0.074	0.075	0.014
EN2	0.104	0.176	**0.694**	−0.055	−0.062	0.232	−0.019
EN3	0.122	0.098	**0.835**	−0.114	−0.005	0.097	0.008
EN4	0.114	0.011	**0.795**	−0.059	0.093	0.084	0.081
DE1	0.000	0.046	−0.109	**0.822**	−0.018	0.080	−0.121
DE2	−0.022	0.078	−0.102	**0.850**	0.049	0.031	−0.114
DE3	−0.049	0.084	−0.012	**0.759**	0.038	0.059	−0.035
DE4	0.033	−0.034	−0.043	**0.748**	0.111	0.010	0.036
DE5	−0.052	−0.019	−0.084	**0.736**	0.042	0.034	−0.199
IB1	0.147	−0.052	0.052	0.093	**0.824**	0.164	−0.156
IB2	0.062	0.031	0.061	0.059	**0.861**	0.205	−0.177
IB3	0.166	0.017	−0.001	0.084	**0.837**	0.233	−0.114
MA1	0.057	0.120	0.142	0.056	0.149	**0.661**	−0.143
MA2	0.063	−0.020	0.107	−0.017	0.067	**0.739**	−0.007
MA3	0.093	−0.016	0.135	0.019	0.112	**0.756**	−0.077
MA4	−0.029	0.055	0.021	0.100	0.111	**0.802**	−0.153
MA5	−0.008	0.073	0.069	0.070	0.145	**0.724**	−0.135
SC1	−0.029	−0.092	0.053	−0.124	−0.013	−0.169	**0.782**
SC2	0.070	−0.044	0.018	−0.127	−0.125	−0.181	**0.771**
SC3	0.022	−0.067	0.052	−0.099	−0.098	−0.106	**0.830**
SC4	0.073	0.093	−0.036	−0.050	−0.195	−0.035	**0.763**

ASMU, active social media use; PSMU, passive social media use; EN, enjoyment; DE, depression; IB, impulse buying; MA, materialism; SC, self-control.

In addition, there was no problem of multicollinearity in this study, because the variance inflation factors of all constructs were between 1.116 and 1.282, which is well below the threshold of 5 (Hair et al., 2011). In order to overcome common method bias (CMB), some remedies were employed in this study. First, we informed the subjects of the purpose of this study and the confidentiality and anonymity of the questionnaire data (Akhtar et al., 2022a). Second, gender, materialism, and self-control were added as moderators in this study because moderators contribute to the reduction of CMB (Aslam et al., 2021; Akhtar et al., 2022b). Moreover, according to Harman’s single-factor test, the first factor in this research explained 11.5% of the total variance; this was below the 50% threshold (Podsakoff et al., 2003), which indicates that there was no problem of CMB in this study.

### Structural model

The structural equation modeling results demonstrated a good model fit by absolute fit indices (χ^2^/df = 2.412 < 3; RMSEA =0.054 < 0.08) and incremental fit indices (CFI = 0.928 > 0.9; GFI = 0.919 > 0.9; TLI = 0.917 > 0.9; IFI = 0.928 > 0.9).

The proposed hypotheses were examined by evaluating the structural model. The results showed that active and passive social media use significantly and positively affected users’ enjoyment (β = 0.242, *p* < 0.001; β = 0.185, *p* < 0.001), which supported H1 and H2a. Passive social media use significantly and positively affected users’ depression (β = 0.082, *p* < 0.05), which supported H3b. Enjoyment (β = 0.209, *p* < 0.01) and depression (β = 0.28, *p* < 0.001) significantly and positively affected users’ impulsive buying, which supported H3 and H4.

Of the two control variables, only monthly expenditure (β = 0.096, *p* < 0.05) was significantly and positively associated with users’ impulse buying (see Table 4). After adding the two control variables, all research hypotheses were still supported, which indicates that the control variables did not bias the results of this study.

**Table 4:** Path analysis results.

Hypotheses	Standardized (**b**)	*P*-value	Results
H1: ASMU → EN	0.245	0.000^***^	Supported
H2a: PSMU → EN	0.185	0.000^***^	Supported
H2b: PSMU → DE	0.082	0.046^*^	Supported
H3: EN → IB	0.209	0.005^**^	Supported
H4: DE → IB	0.280	0.000^***^	Supported
Expense per month	0.096	0.011^*^	
SM time spent per day	0.058	0.124	

ASMU, active social media use; PSMU, passive social media use; EN, enjoyment; DE, depression; IB, impulse buying.

***p < 0.001; **p < 0.01; and *p < 0.05.

### Mediating and moderating effects

#### Mediating effects

We tested the mediation model using model 4 of the PROCESS macro and the bootstrapping method (with bootstrap resamples *N* = 5,000) (Hayes, 2013). This method has been widely used in many mediating and moderating effects tests (Cai et al., 2021; Van Tran et al., 2022). At the 95% confidence level, the confidence intervals (CIs) did not contain 0, which indicates that the indirect effect was significant.

As shown in Table 5, the indirect effect of active social media use on impulse buying was significant for the mediating variable of enjoyment (β = 0.021, CI = 0.001–0.071), and the CIs did not contain 0. Thus, H5 was supported. The direct effect was also significant (β = 0.285, *p* < 0.001), which revealed a partial mediating role of enjoyment in the relationship between active social media use and impulse buying.

**Table 5:** Mediating effect test results.

Path	Coefficients	*P*-value	95% confidence interval		Results
			Lower	Upper	
Direct effect					
ASMU → IB	0.288	0.000^***^			
PSMU → IB	−0.012	0.787			
Indirect effect					
ASMU → EN → IB	0.021		0.001	0.071	H6 supported
PSMU → EN → IB	0.035		0.015	0.066	H7a supported
PSMU → DE → IB	0.02		0.002	0.044	H7b supported

ASMU, active social media use; PSMU, passive social media use; EN, enjoyment; DE, depression; IB, impulse buying.

*** *p* < 0.001.

The indirect effects of passive social media use on impulse buying were significant for both the mediating variables of enjoyment (β = 0.043, CI = 0.007–0.09) and depression (β = 0.032, CI = 0.003–0.074), which excluded the value of zero. However, the direct effect was not significant (β = 0.079, *p* > 0.05), which indicates that enjoyment and depression fully mediated the relationship between passive social media use and impulse buying. Therefore, H6a and H6b were supported.

#### Moderating effects

We tested the moderation model using model 1 of the PROCESS macro and the bootstrapping method (with bootstrap resamples *N* = 5,000) (Hayes, 2013). Gender did not significantly moderate the effects of either enjoyment (*F* = 0.066, *p* = 0.798) or depression (*F* = 1.272, *p* = 0.26) on impulse buying. Therefore, H7a and H7b were rejected.

**Figure 2:**
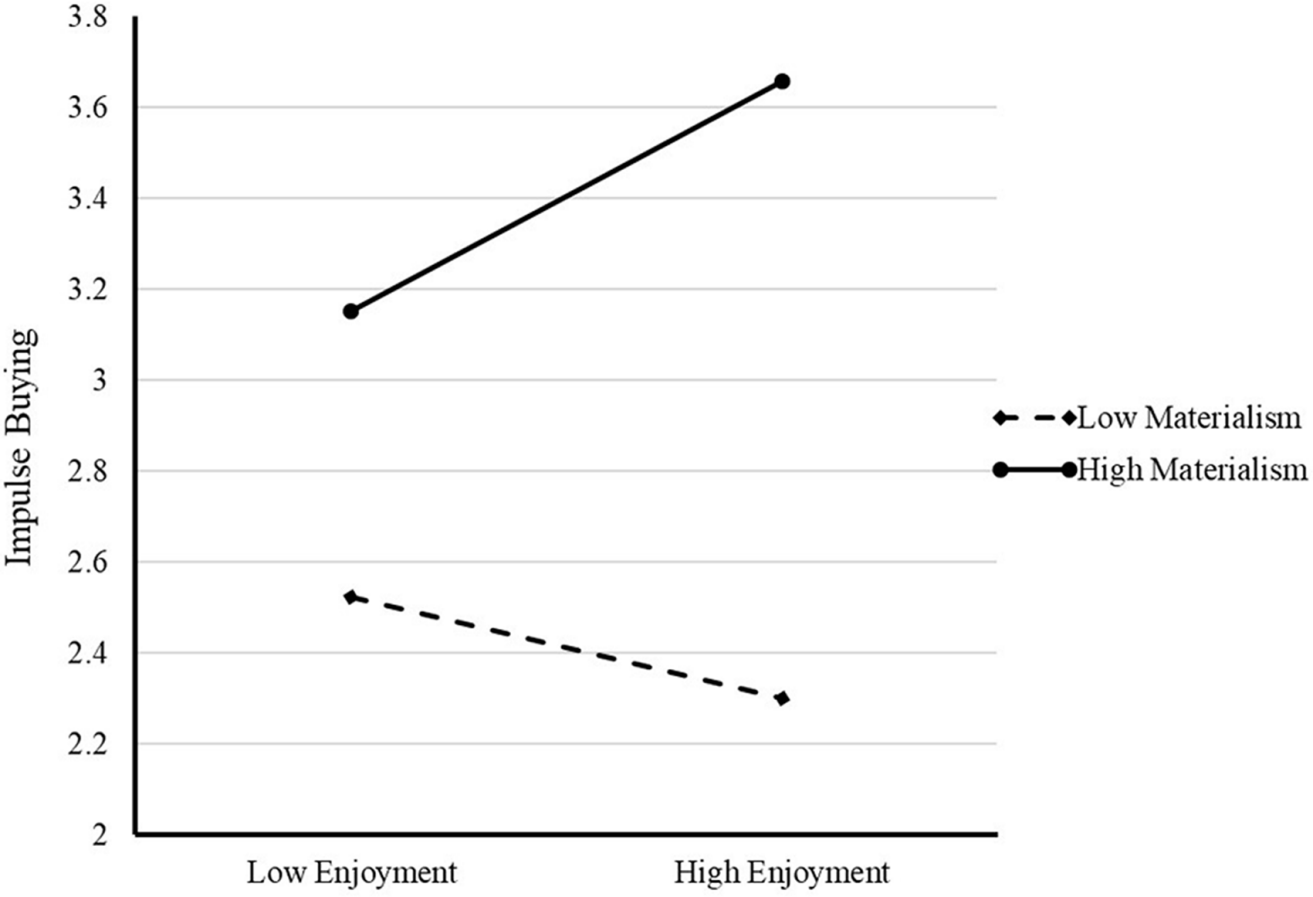
Materialism moderates the relationship between enjoyment and impulse buying (H8a).

Materialism significantly and positively moderated the effect of enjoyment (*F* = 9.473, *p* = 0.022) on impulse buying (Figure 2), which supported H8a. However, materialism did not significantly moderate the association between depression and impulse buying (*F* = 3.83, *p* = 0.051). Therefore, H8b was rejected.

**Figure 3:**
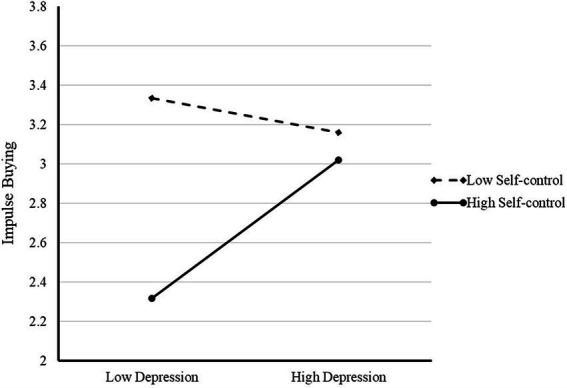
Self-control moderates the relationship between depression and impulse buying (H9b).

Self-control did not significantly moderate the effect of enjoyment on impulse buying (*F* = 2.391, *p* = 0.123). Therefore, H9a was rejected. Consistent with H9b, self-control significantly and negatively moderated the association between depression and impulse buying (*F* = 11.297, *p* = 0.001) (see Figure 3).

## Discussion and conclusion

The purpose of this study was to explore the relationships between different social media use patterns and impulse buying in Chinese college students, as well as the differential emotional mediators and personality trait moderators. We collected data through an online survey and tested the proposed hypotheses and research model using the structural equation modeling method. The results revealed that users’ active and passive social media use were positively associated with their enjoyment, and passive use positively predicted depression. Our results also indicated that both enjoyment and depression can trigger users’ impulse buying. Furthermore, there was an inhibitory effect of self-control on depression-driven impulse buying, and an enhancement effect of materialism on enjoyment-driven impulse buying. The present results have important implications for understanding the mechanisms underlying impulse buying in the context of social media and guiding rational online consumption.

The results indicated that different patterns (active and passive) of social media use had different emotional impact mechanisms on users’ impulse buying. Active social media use increased users’ enjoyment (H1), whereas passive browsing boosted users’ depression (H2b), which is consistent with previous research (Verduyn et al., 2017; Chen S. et al., 2019; Valkenburg et al., 2022). The findings from this study confirm our contention that passive social media use significantly enhances users’ enjoyment (H2a), which further supported the “emotional contagion phenomenon” proposed by Lin and Utz (2015). Given that social media is flooded with positive statuses, users can experience contagion and delight by these when browsing social media, even when they are not interacting with other people. Notably, in agreement with our expectations and prior research (Escobar-Viera et al., 2018; Valkenburg et al., 2022), the association between active use and depression was not significant (β = −0.075, *p* = 0.08). Our research findings support hypotheses H3 and H4 that enjoyment and depression are positively and significantly associated with social media users’ impulse buying, which is in line with previous results (Liu et al., 2019; Zhao et al., 2021). When individuals feel pleasure and enjoyment in social media use, they may have the urge to make an impulse purchase; however, they can also feel the urge to impulse buy when they feel depressed, sad, or unhappy.

The main findings of the study revealed the mediating effects of enjoyment and depression between different social media use and impulse buying. Enjoyment significantly partially mediated the relationship between active social media use and impulse buying (H5). Users actively interact with other people on social media (e.g., by chatting, liking, and commenting), perhaps because they are interested in a specific product, or due to recommendations from social media friends. These active interactions could make them feel happiness and enjoyment, and promote impulse purchases. Other scholars have proposed a similar association (Chen Y. et al., 2019). Furthermore, passive social media use significantly and indirectly predicted users’ impulsive consumption *via* the mediating effects of enjoyment and depressive mood (H6), but not directly. This insignificant direct effect between passive browsing and impulse buying aligns with Leong et al. (2018) results, and may be because enjoyment and depression have full mediating effects. These results indicate that when users browse many positive posts on social media, they are likely to make impulse purchases only when they are experiencing enjoyment or feeling depressed.

Gender was found to be a non-significant moderator of the relationships between enjoyment, depression, and impulse buying (H7a/b). The results were inconsistent with those of Atulkar and Kesari (2018), who found that gender positively moderates the relationship between enjoyment and impulsive consumption. This contradictory result may be because the participants in this study were young people, who engage in more impulse buying than older people (Styvén et al., 2017). Compared with demographic factors, psychological factors may be more influential in triggering impulse purchase behaviors in social commerce consumers (Leong et al., 2018).

As predicted, materialism significantly positively moderated the relationship between enjoyment and impulse buying in social media users (H8a). Atulkar and Kesari (2018) indicated that enjoyment and materialism positively predict impulsive consumption. The findings of the present study further extended the existing literature by verifying the reinforcing effect of materialism on the positive relationship between enjoyment and impulse buying. Individuals with high materialism were more likely to consume impulsively to satisfy their material desires and obtain more happiness when they are experiencing enjoyment on social media. Contrary to hypothesis 8b, we found no significant moderating effect of materialism in the relationship between depression and impulse buying. One possible explanation for this phenomenon is that consumers’ negative emotions can prompt similar negative valence memories, which renders them unable to think systematically and make rational judgments (Raghunathan and Pham, 1999), thereby shaping impulse buying decisions. This process occurs in both high materialists and low materialists, with no significant difference between the two.

Another interesting finding is the negative moderating effect of self-control on the association between depression and impulse buying (H9b). Other scholars have proposed a similar association (Li et al., 2021). Young people with weaker impulse control are more inclined to spend money impulsively to cope with their negative affect. This is in line with previous findings that people with low self-control are more likely to exhibit impulsiveness, which is driven by negative emotion (Loewenstein, 1996). However, we found no such significant moderating effect of self-control in the relationship between enjoyment and impulse buying (H9a). When people feel enjoyment during social media usage, they buy products on impulse, and there is no significant difference between those with high self-control and those with low self-control. The results are consistent with earlier research (Balleyer and Fennis, 2022), which claimed that positive emotions lead to less vigilance and thereby reduce self-regulation. When individuals experience enjoyment, those with high self-control are not significantly different from those with low self-control in their ability to control impulsive spending.

## Implications and limitations

### Theoretical implications

Several valuable theoretical implications have been put forth by this study. Prior research has examined direct associations between social media use and impulsive purchase (Leong et al., 2018; Pellegrino et al., 2022), with a lack of consideration of different social media usage patterns and mediating effects. This research expands the impulse buying literature by developing a model of the relationship between active and passive social media use, emotion, and impulse buying behavior. The present study also adds to the body of knowledge on how emotions of different valences (enjoyment and depression) mediate the association between active and passive social media use and impulsive consumption behavior. It is noted that this study finds a partial mediating effect of enjoyment on the relationship between active social media use and impulse buying, and a full mediating effect of enjoyment and depression between passive social media use and impulsive buying. Finally, the moderating effects of self-control and materialism on the relationship between emotions and impulse buying were explored, which is uncommon in the current research on impulsive consumption. As a result, this study proposes a thorough mediated and moderated model to investigate the effect of active and passive social media use on impulse buying.

### Practical implications

The study findings also have valuable implications for young consumers, social media platform providers, and government practices. Young people spend a lot of time using social media, and although browsing social media can increase enjoyment, it also increases the risks of depression and impulse buying. Comparatively, actively interacting with others (e.g., chatting, liking, and commenting) or actively posting status updates on social media can enhance positive emotion. It could be useful to educate young users about ways to connect with other people online, such as interacting with friends as well as strangers, that are better for their mental health. However, when interacting with friends, users should be sensible and vigilant about recommendations from friends to avoid impulsive and excessive consumption. Built on the direct impact of active social media use on impulse purchases, social media platform providers should track cookies to identify the items users mentioned in their interactions (e.g., comments or likes) and time spent completing a transaction. This information can be used in the design of algorithms for personalized content or advertisement push, which may increase social media users’ consumption. According to the emotional contagion phenomenon confirmed in this study, browsing positive content posted on social media can enhance users’ positive emotions and impulse buying. The Chinese government should strive to formulate laws, regulations, and related policies to strengthen the supervision of content posted on social media platforms in order to maintain more positive content and eliminate harmful information, which is beneficial to expand consumption.

Both enjoyment and depression may increase impulsive consumption, but young people with poor self-control should be wary of unhealthy impulsive purchases when they are depressed. College students should consciously improve their self-control, which can reduce irrational consumption caused by depression. Materialists should be wary of irrational consumption when they are feeling enjoyment on social media. Schools and universities should increase communication and lectures to guide young people to establish the correct view of money and values. In addition, the government should consider posting content on its official homepage of social media (e.g., Weibo or their WeChat Official Account) calling on young social media users to reduce materialism and maintain rational consumption. Therefore, our findings that emotion mediated, and personality traits moderated, the relationship between social media use and impulse buying have implications for interventions that target college students’ healthy use of social media and rational consumption.

### Limitations and future research direction

This study has several limitations. First, this study has some methodological limitations. This was a cross-sectional study, which makes it difficult to infer causal relationships between social media use and impulse buying. Future experimental or longitudinal studies could further verify the results of this research. Second, our results are limited to Chinese college student samples, and care should be taken when generalizing our findings to other age groups, other social media, or other countries. Third, active social media use can be further divided into active social use and active non-social use, according to previous studies (Gerson et al., 2017). Future research could explore the underlying mechanisms in greater depth by subdividing these dimensions. Finally, the effect size of passive use on depression and the explanatory R-squares of enjoyment, depression, and impulse buying were small in this study. Additional mediating variables, such as envy and anxiety, should be explored in future work.

## Data availability statement

The raw data supporting the conclusions of this article will be made available by the authors, without undue reservation.

## Ethics statement

Ethical review and approval was not required for the study on human participants in accordance with the local legislation and institutional requirements. The patients/participants provided their written informed consent to participate in this study.

## Author contributions

SC: conceptualization, methodology, and writing a draft of the manuscript. KZ: reviewing and editing. YC: reviewing and supervising. All authors contributed to the article and approved the submitted version.

## Funding

This research was funded by Chongqing Office for Social Sciences Planning (Grant No. 2020BS33), Chongqing Technology and Business University (Grant No. 1955071), Fundamental Research Funds from the Central Universities (Grant No. 2022CDJSKPY14), and National Social Science Foundation Research Program (Grant No. 21BSH117).

## Conflict of interest

The authors declare that the research was conducted in the absence of any commercial or financial relationships that could be construed as a potential conflict of interest.

## Publisher’s note

All claims expressed in this article are solely those of the authors and do not necessarily represent those of their affiliated organizations, or those of the publisher, the editors and the reviewers. Any product that may be evaluated in this article, or claim that may be made by its manufacturer, is not guaranteed or endorsed by the publisher.

## Appendix A

**Table:**

Constructs	Items	
Active social media use (Gerson et al., 2017; Chen S. et al., 2019)	ASMU1	Posting status updates on social media
	ASMU2	Posting photos on social media
	ASMU3	Posting videos on social media (delete)
	ASMU4	Chatting or interacting with others on social media
	ASMU5	Liking others’ statuses, pictures, etc
	ASMU6	Commenting on others’ statuses, pictures, etc
Passive social media use (Gerson et al., 2017; Chen S. et al., 2019)	PSMU1	Browsing others’ statuses passively (without liking or commenting on anything)
	PSMU2	Browsing others’ pictures or videos passively (without liking or commenting on anything)
	PSMU3	Browsing others’ homepages (delete)
	PSMU4	Checking to see what others are up to (delete)
Enjoyment (Heijden, 2003; Gan and Li, 2018)	EN1	I find social media to be entertaining overall
	EN2	Using social media is an agreeable way of passing time
	EN3	I find using social media to be pleasurable overall
	EN4	I find that using social media makes me enjoy
Depression (Tandoc et al., 2015; Chen S. et al., 2019)	DE1	I felt depressed
	DE2	I felt fearful
	DE3	I felt lonely
	DE4	I felt sad
	DE5	I thought my life had been a failure
Impulse buying (Li et al., 2020)	IB1	I always have the impulse to have a product immediately when I see it on social media
	IB2	I always have the impulse to buy some unplanned products that I did not intend to buy
	IB3	I have an impulse to consumption on social media
Materialism (Li et al., 2020)	MA1	I admire people who own expensive homes, cars, and clothes
	MA2	The things I own say a lot about how well I’m doing in life
	MA3	Buying things gives me a lot of pleasure
	MA4	I’d be happier if I could afford to buy more things
	MA5	My life would be better if I owned certain things I do not have
Self-control (Luo et al., 2021a)	SC1	I do things that bring me happiness but are harmful to me (R)
	SC2	Sometimes I get distracted by having fun and cannot finish the task on time (R)
	SC3	Sometimes I cannot help but do things that I know are wrong (R)
	SC4	I often act without thinking (R)

“R” means this item was reverse coded.
